# Teaching and Learning Core Values in General Practice/Family Medicine: A Narrative Review

**DOI:** 10.3389/fmed.2021.647223

**Published:** 2021-05-11

**Authors:** Nele R. M. Michels, Roar Maagaard, Igor Švab, Nynke Scherpbier

**Affiliations:** ^1^Department of Family Medicine and Population Health, Faculty of Medicine and Health Sciences, University of Antwerp, Antwerp, Belgium; ^2^Centre for Health Sciences Education, Aarhus University, Aarhus, Denmark; ^3^Department of Family Medicine, Faculty of Medicine, University of Ljubljana, Ljubljana, Slovenia; ^4^Department of Primary and Community Care, Radboud University Nijmegen Medical Center, Nijmegen, Netherlands

**Keywords:** general practice, family medicine, core values, medical education, person-centered care, continuity of care, cooperation in care, community-oriented care

## Abstract

General Practice/Family Medicine (GP/FM) is a key discipline within primary health care and so by extension for the whole health care system. An essential condition for effective GP/FM care is a work force that is highly qualified. As society is changing rapidly, a revision of the GP/FM definition is ongoing, in addition to a recent movement of identifying related core values. In this paper, we want to give an overview on how these new paths and perspectives are currently reflected in GP/FM teaching and training. We selected four core values that fit in with possible future visions: person-centered care, continuity of care, cooperation in care, and community-oriented care. By a narrative review, we observed that GP/FM education toward core values is often built around overarching topics. Teaching and learning take place in specific contexts, most of all through placements within communities, primary care settings, or hospital wards. Mixed teaching- and training methods are used combining knowledge, skills, and attitude. Furthermore, collaboration with other health professionals and peers is stressed, in addition to the importance of role models, a holistic focus and the involvement of patients. Since these core values are important within GP/FM and rather few studies on the educational aspects and learning tools were found we advocate encouraging each other more to share good practices, certainly the innovative ones specifically related to GP/FM.

## Introduction

In 1978, the Alma Ata declaration (WHO) recognized primary health care as the important first contact of the population for their health care, providing preventive, curative, and rehabilitation services for the community (1). This has been re-affirmed in 2018 (2). General Practice/Family Medicine (GP/FM) is a key discipline within primary health care and so by extension for the whole health care system. The WONCA Europe consensus document on the European definition of GP/FM (2002, with revisions in 2005 and 2011) states that “GP/FM is an academic and scientific discipline, with its own educational content, research, evidence base and clinical activity, and a clinical specialty orientated to primary care” (3). An essential condition for effective GP/FM care is a work force that is highly qualified. In order to achieve this, European specialty training centers for GP/FM can rely on European legislation (4) and on the EURACT European Training Requirements (5, 6). The latter is a recently drafted guideline based on the European definition of GP/FM and the 12 characteristics, six core competences, and three features as expressed by the well-known WONCA tree (7). However, society is changing rapidly, and so the organization and objectives of health care. General Practice/Family Medicine is strongly developing worldwide, both in a conceptual and in a technical way (8, 9). The convergence of medicine, psychology, sociology, and technology leads to the achievement of care that is predictive, preventive, personalized, and participatory (8, 10, 11).

A pan-European movement toward a revision of the GP/FM definition is ongoing, in addition to a recent movement of identifying related core values. A WONCA Europe Task Force is currently developing a supported European vision, which was presented on the annual WONCA Europe Conference (Berlin/online December 2020). Also, previous activities to establish new core values have already been rolled out in the Netherlands (12) and the Scandinavian countries (13). Revising the GP/FM definition and defining core values will help to better clarify (the importance of) the discipline of GP/FM. This will lead to new core competencies and core tasks for General Practitioners/Family Doctors (GPs/FDs) as well. In the coming years, we will have to secure that future GPs/FDs incorporate these core values in their behavior.

In this paper, we want to give an overview on how these new paths and perspectives are currently reflected in GP/FM teaching and training. Pending the final results of the European vision on core values, we selected four core values that fit in with possible future visions and are based on the thinking that has already been done (12, 13): (1) person-centered care, (2) continuity of care, (3) cooperation in care, and (4) community-oriented care. We questioned how those four values are currently taught in GP/FM oriented training. As a result, good practices will be shown so that they can serve as inspiration.

## Methods

We chose to write a narrative review on educational activities with respect to these four core values in order to present the current yet broad perspective (14, 15). We searched for relevant literature and published reports, that cover the four selected core values and their translation into GP/FM education. From the literature we chose the elements that illustrated how we could translate the prevailing discussion on core values in GP/FM to teaching and learning.

Main keywords for the search (PubMed) included the four selected core values (“person-centered care,” “continuity of care,” “cooperation in care,” and “community-oriented care”) in combination with “education or training” and “general practice” or “family medicine.” By the snowball method also other articles or reports were sought. We narrowed the search by selecting only literature in English from the last 10 years. A first screening for relevance for the goal of our study was done by reading titles and abstracts.

Per core value, we summarized and synthesized the findings from the respective articles into a qualitative summary, with references to good practices.

## Results

### Person-Centered Care

Person-centered care can be defined as seeing a patient as a particular person in a particular context with his or her knowledge, desires, values, preferences, lifestyle, and social circumstances and including the total health perspective. As such, patients are equal partners in planning, developing, and monitoring care to make sure the care provided meets their needs, their realistic health-, and life goals (16–19). The concept of person-centered care is rather new, evolving, and complex, emphasizing the need of new ways to deliver care and look at patient–doctor relationships (19, 20). Conceptual frameworks can give practical guidance both in health care and in teaching and training (20, 21).

Our review showed that some studies are specifically dealing with GP/FM education or training to improve person-centered care (22–24). However, in most of the studies retained, person-centered care as a learning objective is usually mentioned together with other topics such as shared decision making, comprehensive approach, empowerment, compassionate care, etc. (25–31). Moreover, person-centered care and these other topics are often seen as indirect objectives to be taught when organizing training programs around themes as dementia, multimorbidity, or clinical care of refugees (22–24). Gillies pleas for medical humanity as an academic discipline and resource for education (32). This could be feeding specific training programs for person-centered care, which are essential as our modern society is volatile, uncertain, complex, and ambiguous (VUCA) (28).

A variety of educational tools, used for person-centered care in GP/FM for both undergraduate students and primary care staff, is described. Willoughby et al. refer to a Longitudinal Family Medicine Experience for first-year medical students (31). Paired to a GP/FD, students encounter patients in the real GP/FM clinical context, writing patient logs, and a reflective essay. To improve health literacy among health professionals an 8 h training–intervention was set up and evaluated in three European countries (30). The training was a combination of knowledge development, practicing communication skills, and skills- and attitude building e.g., around self-management. The participants perceived the training as valuable, although a rather low number of GPs/FDs participated. It is striking that in quite some settings “art” is used as an educational tool; movies, television programs, books, and theater can be instructive sources to enhance knowledge, skills, and attitude related to person-centered care (25, 27, 29, 32). General Practice/Family Medicine teachers can be inspired and helped on their way by offered templates for “Cinemeducation” (25) or by 12 tips to use the Applied Improvisation method (29).

### Continuity of Care

Although GPs/FDs often with pride praise both their personal profession and their specialty for delivering continuity of care for patients there are remarkably few research papers on how to teach this to medical students and to GP/FM trainees.

Students have most often a very short stay in GP/FM, so they are not able to get a personal learning experience of how this continuity is of value for both the patient and the healthcare professional. Specific teaching methods have been explored, and the use of standardized patient encounters has shown to be useful in teaching continuity relationships as well as chronic disease management (33). Especially for patients with chronic conditions a longitudinal relationship with their doctor is beneficial. In that respect, Preci et al. suggested a model where students “adopt” a chronic patient for a period (34). They showed that this method enabled students to practice more empathetic medicine in the future and to improve the skills needed in a complex relationship.

General Practice/Family Medicine trainees have longer clinical training periods and hence clinical work and training there gives them the opportunity to experience the value and challenges of continuity of care. In a study of Delva et al. trainees valued the efficiency gained by knowing patients and they felt rewarded of being recognized by patients (35). Also supplementary methods can be used in a GP/FM training setting; Sternlieb (2012) showed how a 1-year program with a once-a-month case conference format on long-term healing relationship can help a GP/FM trainee in managing the many health issues a patient might present over time (36).

Relating to both students and GP/FM trainees the importance of exposure to good GP/FM role models must be emphasized. Part of being a good role model often implies storytelling about patients that actually made a difference for the storyteller. Saultz (2016) highlights the importance of storytelling and stresses that meaningful doctor-patient relationships are built on shared experiences (37). Therefore, we must allow such experiences to happen. Moreover, sharing such real stories is seen as an important stimulus for recruitment to GP/FM specialty (37). But how to learn to tell stories? Ventres and Gross (2016) give a very detailed recipe for learning to become a storyteller in GP/FM (38).

### Cooperation in Care

In an aging society where the number of patients with multimorbidity is rising, the number of health professionals around one patient is often more than one. This increases the risk of fragmentation of care and lack of continuity of care, which is not only inconvenient for the patient, but also comes with risks. The transitions between primary and secondary care for example, are associated with an increased risk of medical errors (39). Effective interprofessional collaboration is important to provide effective person-centered care, across the care continuum. Integration of care should be realized at the micro- (clinical integration), meso- (professional and organizational integration), and macro- (system integration) level (40). In this manuscript, we will focus on the micro level: interprofessional collaboration in daily care around a patient.

Interprofessional collaboration is not easy. Professionals experience barriers around the awareness of one another's roles and competences, and around confidentiality, responsibility, and team building (41). Interprofessional education can contribute to better interprofessional collaboration and patient outcomes (42).

When designing interprofessional education the following can be taken into account: involving patients in the design and delivery, providing a holistic focus, focusing on practical actions, deploying multi-modal learning formats and activities, evaluating formative and summative aspects, and encouraging team-based working (43).

There are interesting examples of interprofessional education in a community setting, where students from various health professions and social care experience the value of interprofessional collaboration (44–47).

Within a primary care health center often GPs/FDs will collaborate with nurses. Although learning with and about each other is judged as important, this is not often happening unless someone in the practice is taking the lead in facilitating this (48). The same counts for interprofessional learning between nurses and GP/FM trainees (48). There are enough chances to learn at the workplace while working together, but time for reflection should be created (49). Special effort is needed like in an example of interprofessional education around frail elderly in primary care, where GPs/FDs redefined division of tasks and responsibilities (50).

Collaboration between primary and secondary care doctors is called intraprofessional collaboration. Trainees in both primary and secondary care need to develop competencies to let patients feel that care and information transfer between the medical specialist and the primary care doctors show consistency (51). It is not easy to make primary and secondary trainees meet each other, due to difference in workplace with travel distance and busy rotations. In a project where they met in a digital way, GP trainees could consult internal medicine trainees about patients. This proved to be a rich learning opportunity (52). Also, the moments that primary care trainees have their hospital placement, can be used to learn to collaborate intraprofessionally (53).

### Community-Oriented Care

Community orientation is a concept introduced into GP/FM in order to describe the responsibility of the discipline not only for the individual patient, but also for the community. It implies that the GP/FD needs to be aware of the environment in which he/she practices and to take action in that respect, because community involvement is necessary for the benefit of patients. Future doctors must therefore be aware of the theoretical concepts of public health and participate in some community-based activities, most frequently in health promotion and disease prevention. The concept is strongly promoted by the WHO (54) and involves introducing some concepts of public health into the discipline of GP/FM.

Teaching programs are often based on complex principles of community oriented primary care, developed for the purpose of the course and adapted to the specificities of GP/FM (55, 56). Typically, teaching methods include a theoretical introduction to principles of public health (57), combined with placing the learners in local communities where they participate in the work of a community health center (58). While learning in the community, learners quite often have to perform specific tasks (59). In a lot of these courses the practical part involves participation in regular activities within the community, usually disease prevention and health promotion (60).

Evaluation of these activities shows that the satisfaction with these placements by the students can be very positive, because students value active participation in the community (61), but there are also examples where students are not so satisfied with them mainly due to problems in logistics and because they do not see the relevance of the subject they have to participate in (62). The proper organization of these placements and adequate preparation for the practical tasks seems the key factors of success.

## Discussion

Currently, a European movement towards a new GP/FM definition is going on. In this new definition, new core values will also be included. With this narrative review we were looking for educational practices that address those “candidate” GP/FM core values. Therefore, we selected four core values: person-centered care, continuity of care, cooperation in care, and community-oriented care.

Medical education oriented to GP/FM can be offered on three levels, namely the undergraduate level (or BME, Basic Medical Education), the postgraduate level (or ST, Specialty training), and the GPs/FDs' education (or CME, Continuing Medical Education). All three levels contribute to a strong and trained GP/FM workforce. If we expect, in this changing society, an updated GP/FM definition and related core values, GP/FM teaching and training should be adapted accordingly.

Leaning toward professionalism and humanism, core values do ask for a specific approach toward learning and teaching. Although teaching principles and recommendations are described in literature on medical education in general (63, 64), no consensus on the most effective approach is reached yet (63, 65). Branch describes a combined methods model, including theory as well as practice which are interrelated (65). More in detail, aspects as deliberate practice, experimental learning, feedback and reflection, support by peers in small groups, a longitudinal cohesive program, and a culture of interest in and support for the student are used (66). This is in line with those educational practices in GP/FM that were found and discussed in this review, related to all four core values.

We observed that GP/FM education toward the core values is often built around overarching topics like dementia, multimorbidity, prevention and health promotion, migration, etc. Teaching and learning takes place in specific contexts, most of all through placements within communities, primary care settings, or hospital wards. Mixed teaching- and learning methods are used that combine knowledge building, practicing skills, and a focus on attitude. Moreover, collaboration is stressed as well as with other health professionals as with peers, providing small but safe learning environments. When developing GP/FM education around core values it is also very interesting to be aware of the importance of role models, to keep a holistic focus in mind and to involve patients, both in the design of the training and within the training itself.

We noticed that our search yielded more articles on the core values themselves, rather than on the educational aspects and learning tools. A possible explanation is the fact that we are talking about a young field of teaching. Recognition of GP/FM as a “specialty” among the others just started in the 1960s and 1970s but is still ongoing in some countries around the world (67, 68). Consequently, this is certainly true for the tradition of offering GP/FM education in the medical schools. Even today, it can be a struggle to give GP/FM education sufficient attention, to have it recognized, and to have sufficient and capable GP/FM staff in separate GP/FM departments within the medical institutions (67). So logically, as GP/FM is a rather young discipline with a young field of teaching, it is also a young field of research both on GP/FM itself and on GP/FM teaching. Besides, we have to be aware that there will be a lot of good practices that have not been written about.

That brings us to our recommendations for the future. We should encourage each other more to share good practices, certainly the innovative ones specifically related to GP/FM. Moreover, GP/FM teachers and trainers could evaluate and investigate what works and what does not and (try to) publish this research. If we want to raise GP/FM and GP/FM education to the highest standards, this is essential. This is not an easy job: GP/FM is a specialism with great complexity, GP/FM doctors often have to combine their research activities with ongoing teaching activities and patient care, there often is a lack of direct benefits for research. Therefore, we need to focus on solutions such as collaboration in academic GP/FM departments, involvement of young researchers or students, time-efficiency and the believe that sharing ideas has mutual benefits (69). Most (GP/FM-oriented) medical educational journals have special formats to shortly report on good practices, however, original research articles are needed too.

With this narrative review we wanted to set an example to give guidelines concerning GP/FM education towards core values. By the way, inspiring, stimulating, and empowering each other is something we know as GPs/FDs from our patient care. Let us apply that to our teacher community as well.

We acknowledge some limitations of this narrative review. At the moment of preparing the manuscript a new European-wide approved GP/FM definition is not available yet, neither are clear and defined core values. Therefore, we relied on two regional initiatives, namely the Dutch and the Scandinavian ones (12, 13). Furthermore, as we performed a narrative review, not all research related to this topic is discussed. We aimed to find some good practices and overall views around core values and GP/FM education, so our search was not systematically performed, following a certain search strategy (14, 15).

In conclusion, this narrative review showed that some good practices on education regarding the core values for GP/FM have already been described in literature. Most of all, a mix of educational tools is used taking into account some essential principles as collaboration, using the clinical (primary care) context and role models, keeping holistic views, and involving patients. As such students, GP/FM trainees and GPs/FDs can achieve the essential knowledge, skills, and attitude to be a GP/FD which is orientated to the core values of their profession. This can be even more strengthened by inspiring each other and thus sharing innovative educational methods.

## Data Availability Statement

The original contributions presented in the study are included in the article/supplementary material, further inquiries can be directed to the corresponding author/s.

## Author Contributions

All authors fully contributed at the manuscript and finally approved it. All authors contributed to the design of the study, the literature search, and the writing.

## Conflict of Interest

The authors declare that the research was conducted in the absence of any commercial or financial relationships that could be construed as a potential conflict of interest.
